# The Effects of Excitatory and Inhibitory Social Cues on Cocaine-Seeking Behavior

**DOI:** 10.3389/fnbeh.2016.00217

**Published:** 2016-11-09

**Authors:** Mark A. Smith, Huailin Zhang, Andrea M. Robinson

**Affiliations:** Department of Psychology, Program in Neuroscience, Davidson CollegeDavidson, NC, USA

**Keywords:** cocaine, cues, discriminative stimulus, rat, self-administration, social

## Abstract

Social partners influence the likelihood of using drugs, developing a substance use disorder and relapse to drug use after a period of abstinence. Preclinical studies report that social cues influence the acquisition of cocaine use, the escalation of cocaine use over time, and the compulsive patterns of cocaine use that emerge during an extended binge. The purpose of this study was to examine the effects of social cues on the reinstatement of cocaine-seeking behavior after a period of abstinence. Male rats were obtained at weaning, assigned to triads (three rats/cage), reared to adulthood and implanted with intravenous catheters. Rats from each triad were then assigned to one of three conditions: (1) test rats were trained to self-administer cocaine and were tested for reinstatement; (2) cocaine partners were trained to self-administer cocaine and were predictive of response-contingent cocaine delivery; and (3) abstinent partners were not given access to cocaine and were predictive of extinction. The test rats alternated social partners every 5 days for 20 days such that responding was reinforced with cocaine in the presence of the cocaine partner (S^+^) for 10 days and not reinforced with cocaine in the presence of the abstinent partner (S^−^) for 10 days. Responding of the test rats was then extinguished over 7 days under isolated conditions. Tests of reinstatement were then conducted in the presence of the cocaine partner and abstinent partner under extinction conditions. Neither social partner reinstated responding relative to that observed on the final day of extinction; however, responding was greater in the presence of the cocaine partner (S^+^) than the abstinent partner (S^−^) during the reinstatement test. These data fail to demonstrate that a social partner reinstates cocaine-seeking behavior after a period of abstinence, but they do indicate that social partners can serve as either excitatory or inhibitory discriminative stimuli to influence drug-seeking responses.

## The Effects of Social Cues on Cocaine-Seeking Behavior

Social peers influence the likelihood of using drugs (Bahr et al., 2005), the likelihood of developing a substance use disorder (Scherrer et al., 2008; McCutcheon et al., 2013), and the likelihood of relapse after a period of abstinence (Winters et al., 2000; Chung and Maisto, 2006). Recently, preclinical studies have begun examining the influence of social peers on drug self-administration and other measures of drug-seeking behavior (see reviews by Neisewander et al., 2012; Bardo et al., 2013; Zernig et al., 2013; Strickland and Smith, 2014, 2015). We recently reported that cocaine intake is facilitated in the presence of a social partner that is also self-administering cocaine, but cocaine intake is inhibited if a social partner is abstinent (i.e., if the partner does not have access to cocaine). Moreover, these effects are generally consistent across assays measuring the acquisition of cocaine use, the maintenance of cocaine use, and the escalating and excessive patterns of cocaine use that emerge over time (Smith, 2012; Smith et al., 2014; Robinson et al., 2016).

Preclinical studies are beginning to examine the role of social context in the reinstatement of drug-seeking behavior. Reinstatement procedures model relapse to drug use after a period of abstinence by first establishing drug self-administration, then extinguishing responding by removing access to the drug, and then reinstating responding by providing an excitatory stimulus. For instance, excitatory contextual cues (e.g., odor, white noise) that predict cocaine availability reinstate responding after a period of extinction, whereas inhibitory contextual cues that predict extinction do not (Weiss et al., 2000; Alleweireldt et al., 2001). Reinstatement of responding can also be induced and modified by social stimuli. For example, social stress caused by social defeat reinstates cocaine-seeking behavior (Manvich et al., 2016) and increases the effects of a cocaine prime (Ribeiro Do Couto et al., 2009). Conversely, the opportunity to interact with a social partner under drug-free conditions can prevent the reacquisition of a cocaine-induced conditioned place preference (Fritz et al., 2011).

Similar to other contextual cues, social partners may serve as discriminative stimuli that predict the availability of drugs and the opportunity for drug use. For instance, an individual that uses drugs and encourages drug use in others may serve as a discriminative stimulus predicting that drug-seeking behaviors will be reinforced (S^+^); however, an individual that practices abstinence and discourages drug use in others may serve as a discriminative stimulus predicting that drug-seeking behaviors will not be reinforced (S^−^). As noted previously, cues that predict cocaine availability reliably reinstate drug-seeking behavior, whereas cues that predict extinction do not (Alleweireldt et al., 2001; Ciccocioppo et al., 2002).

The purpose of the present study was to determine whether a drug-using social partner that predicts response-contingent drug availability (S^+^) reinstates responding to a greater extent than an abstinent partner that does not predict response-contingent drug availability (S^−^). To this end, male rats were implanted with intravenous catheters and trained to self-administer cocaine on a fixed ratio (FR1) schedule of reinforcement. The test rats alternated social partners every 5 days over 20 consecutive days during which they were tested with response-contingent cocaine in the presence of a cocaine partner and tested under extinction conditions in the presence of an abstinent partner. Next, responding was extinguished in test rats under isolated conditions over 7 days. Reinstatement tests were then conducted in the presence of the cocaine partner and the abstinent partner. We predicted that the reinstatement of drug-seeking behavior would be greater in the presence of the cocaine partner than in the presence of the abstinent partner.

## Materials and Methods

### Animals

Male, Long-Evans rats were obtained at weaning (~21 days) and housed in triads (*n* = 36 rats; 12 triads; three rats/cage) in polycarbonate cages (interior dimensions: 50 cm × 28 cm × 20 cm) until surgery and catheter implantation. All subjects were kept in a temperature- and humidity-controlled vivarium on a 12-h light/dark cycle (lights on: 05:00). Except during lever-press training, food was continuously available in the home cage. Fresh tap water was continuously available in the home cage throughout the study. All subjects were maintained in accordance with the Institutional Animal Care and Use Committee of Davidson College and the *Guide for the Care and Use of Laboratory Animals* (Institute of Laboratory Animal Resources (US), 1985).

### Apparatus

Lever-press training was conducted in commercially available operant conditioning chambers from Med Associates, Inc. (St. Albans, VT, USA). These chambers were equipped with a house light, two response levers and a food hopper. Software and interfacing for these chambers were supplied by Med Associates, Inc.

Drug self-administration sessions were conducted in custom-built, operant conditioning chambers (Faircloth Machine Shop, Winston-Salem, NC, USA) described previously (Smith, 2012; Lacy et al., 2014). Briefly, each chamber was equipped with one retractable response lever and an infusion pump mounted outside the chamber. Drug infusions were delivered through a Tygon tube protected by a stainless steel spring and attached to a counterbalanced swivel at the top of the chamber. Response levers, syringe pumps, interfacing and computer software were obtained from Med Associates, Inc., (Fairfax, VT, USA). Chambers used for isolation testing consisted of one 30 cm × 30 cm × 30 cm chamber. Chambers for partner testing were constructed from two isolated chambers, each with one sidewall removed, and connected with a 14-gauge wire screen panel at existing corner supports. The wire screen permitted two rats full visual, auditory, olfactory and limited tactile contact with one another, but prevented one rat from accessing the response lever and infusion lines of its partner. Importantly, these chambers allowed two rats to intravenously self-administer drugs simultaneously in the same chamber.

### Lever-Press Training

Five weeks after arrival and 1 week prior to catheter implantation, rats were food restricted to no less than 85% of their free-feeding body weights and trained to press a response lever on an FR schedule of food reinforcement during daily training sessions. During these sessions, each response was reinforced with a single 45-mg grain pellet (Bio-Serv, Flemington, NJ, USA). Sessions terminated automatically once 40 pellets were delivered or 2 h elapsed, whichever occurred first. Training continued in this manner until a rat earned 40 pellets during any four individual sessions.

### Catheter Implantation

Six weeks after arrival, rats were anesthetized with a combination of ketamine (100 mg/kg, ip) and xylazine (15 mg/kg, ip) and surgically implanted with intravenous catheters into the right jugular vein. Ketoprofen (3.0 mg/kg, sc) was given immediately after surgery and again 24 h later for analgesia. Beginning on the day of surgery, a solution of heparinized saline and ticarcillin (20 mg/kg, iv) was infused through the catheter daily to prevent infection and maintain patency. After 7 days, ticarcillin was discontinued and only heparinized saline was used to maintain catheter patency.

### Behavioral Testing

Immediately after surgery, the three rats of each triad were randomly assigned to one of three conditions: (1) test rats were trained to self-administer cocaine and examined during tests of reinstatement; all primary hypotheses were tested using data from these rats; (2) cocaine partners were trained to self-administer cocaine and served as discriminative stimuli for response-contingent cocaine delivery (S^+^); and (3) abstinent partners never had access to cocaine and served as discriminative stimuli for extinction (S^−^).

Three days following surgery, test rats and cocaine partners were given one session to acquire cocaine self-administration. During this session, rats were placed in chambers alone, isolated from their partners. The session began with a noncontingent infusion of 0.5 mg/kg cocaine (Research Triangle Institute, Research Triangle Park, NC, USA) and the insertion of the retractable lever into the cage. Each response on the response lever produced an infusion of cocaine on an FR1 schedule of reinforcement. Each infusion delivered 0.5 mg/kg cocaine at an 0.05 mg/ml concentration over a duration of 2.0–2.4 s (based on body weight), representing infusion volumes of 0.036–0.043 ml/infusion. Coincident with the infusion, the lever retracted for 20 s to signal a timeout period in which cocaine was not available. After 20 s, the lever was reinserted and cocaine was again available on the FR1 schedule of reinforcement. This initial acquisition session was terminated automatically once 21 infusions were obtained.

Over the next 20 consecutive days, test rats alternated social partners every 5 days. During this period, test rats were tested with cocaine in the presence of their cocaine partner for 10 days (2 cycles × 5 days) and under extinction conditions in the presence of their abstinent partner for 10 days (2 cycles × 5 days). Consequently, the cocaine partner served as a discriminative stimulus for response-contingent cocaine delivery (S^+^) and the abstinent partner served as a discriminative stimulus for extinction (S^−^). During sessions with the cocaine partner, both rats had access to cocaine on an FR1 schedule and no limit was placed on the number of infusions that could be earned. During sessions with the abstinent partner, neither rat had access to cocaine. For the test rat, responses on the lever produced response-contingent infusions of saline on an FR1 schedule and no limit was placed on the number of infusions that could be earned. The order of exposure to the cocaine and abstinent partners (i.e., cocaine partner first vs. abstinent partner first) was counter-balanced across test rats. All sessions were 2 h in duration and began promptly at the beginning of the dark cycle (lights off: 1700).

Over the next 7 days, test rats were tested in isolation under extinction conditions. During these sessions, responses on the lever produced contingent infusions of saline on an FR1 schedule, with no limit placed on the number of infusions that could be earned.

Over the next 2 days, reinstatement tests were conducted in which responding was examined in test rats. Responding was examined in the presence of the cocaine partner one day (the cocaine partner had access to response-contingent cocaine during this session) and in the presence of the abstinent partner on the other day. The order of partner presentation was counter-balanced across test rats. Regardless of partner, responses on the lever by the test rat produced response-contingent infusions of saline (i.e., test rats were tested for reinstatement under extinction conditions).

On days in which cocaine partners were not tested in the presence of a test rat, they self-administered cocaine in isolated chambers under identical experimental conditions (FR1 schedule, 0.5 mg/kg/infusion, 2-h sessions). On days in which abstinent partners were not used for testing, they remained isolated in polycarbonate cages.

### Data Analysis

Data from the first 20 days of testing were examined via three-way (partner × cycle × day), repeated-measures analysis of variance (ANOVA). Data from the following 7 days (i.e., isolation testing) were examined via one-way, repeated-measures ANOVA using day as the factor. Data from the reinstatement tests were examined via dependent-samples *t*-tests using partner/session as the factor (an initial test that included the order of presentation as a between-subjects factor was not significant and thus removed from the analysis). The effect size was calculated via the Cohen’s *d* statistic. All tests were two-tailed and the alpha level was set at 0.05.

## Results

All test rats responded on the first day of cocaine self-administration when they were tested in isolation, and 11 out of 12 test rats received the maximum number of infusions available (*n* = 21 infusions; data not shown).

**Figure 1:**
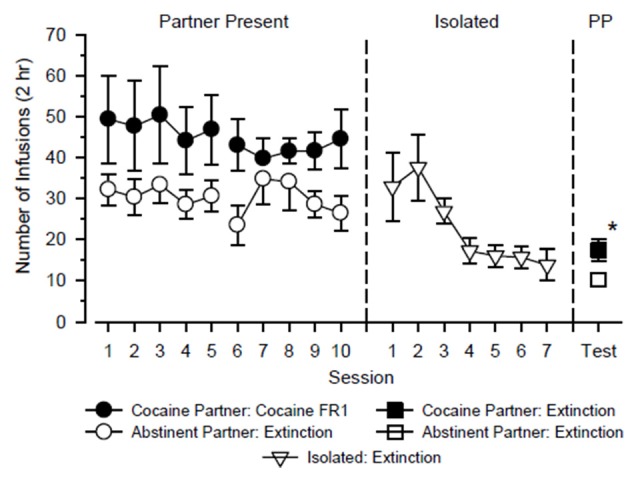
**Responding of test rats (*n* = 12) over the course of the study.** The test rats alternated partners every 5 days over 20 consecutive days in which they were tested with cocaine in the presence of a cocaine partner and tested under extinction conditions in the presence of an abstinent partner (Partner Present: left). Subsequently, test rats were tested in isolation over the next 7 days under extinction conditions (Isolated: center). Finally, reinstatement tests were conducted in test rats over the next 2 days in which extinction responding was measured in the presence of the cocaine partner and the abstinent partner (PP: right). Left axis depicts number of infusions obtained during 2-h test sessions. Vertical lines extending from data points represent the SEM; where not indicated, the SEM fell within the data point. *Indicates significant difference between partner conditions.

The test rats alternated partners every 5 days over 20 consecutive days in which they were tested with cocaine in the presence of a cocaine partner for 10 days (2 cycles × 5 days) and tested under extinction conditions in the presence of an abstinent partner for 10 days (2 cycles × 5 days). A three-way ANOVA using the factors of partner, cycle and day revealed a significant main effect of partner (*F*_(1,44)_ = 4.939, *p* = 0.048), but no other significant main effect or interaction was observed. As shown in Figure 1, test rats responded more when tested with cocaine in the presence of a cocaine partner than when tested under extinction conditions in the presence of an abstinent partner. Responding varied across days and between cycles, but these differences were small and not statistically significant.

Prior to reinstatement testing, responding in test rats decreased in a monotonic fashion when they were tested under extinction conditions in isolation over seven consecutive days (main effect of day: *F*_(6,72)_ = 4.360, *p* = 0.001). Following extinction, neither the presence of the cocaine partner nor the presence of the abstinent partner significantly reinstated responding relative to the final day of extinction (*p* = 0.336 and 0.430, respectively). During the reinstatement tests, responding was significantly greater in the presence of the cocaine partner than in the presence of the abstinent partner (*t*_(12)_ = 2.558, *p* = 0.025), and this difference was characterized by a large effect size (*d* = 1.01).

**Figure 2:**
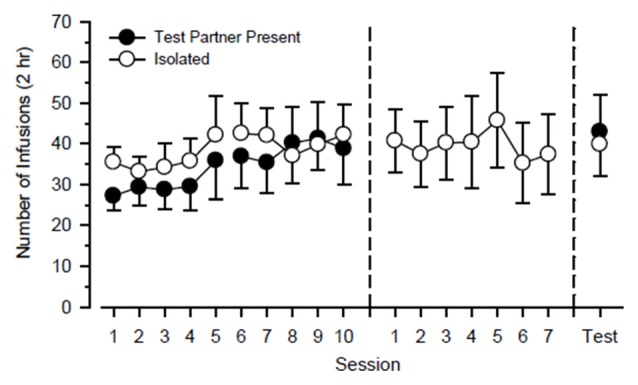
**Cocaine-maintained responding of cocaine partners (*n* = 12) over the course of the study.** Cocaine partners alternated responding in the presence of a test rat and in isolation every 5 days over 20 consecutive days (left). Subsequently, cocaine partners responded exclusively in isolation over the next 7 days (center). Finally, cocaine partners responded over the next 2 days in the presence of a test rat and in isolation (right). Left axis depicts number of infusions obtained during 2-h test sessions. Vertical lines extending from data points represent the SEM.

Figure 2 shows cocaine-maintained responding of the cocaine partner throughout the study. During the first 20 days of the study, a three-way ANOVA using the factors of partner, cycle, and day failed to reveal any significant main effect or interaction. During the next 7 days of the study in which they responded exclusively in isolation, responding was steady and did not vary significantly as a function of day. Responding also did not differ significantly during the final 2 days of the study during which they responded in the presence of the test rat on one day and responded in isolation on the other day.

## Discussion

The principal findings of this study are that: (1) test rats responded more when tested with response-contingent cocaine in the presence of a cocaine partner than when tested under extinction conditions in the presence of an abstinent partner; (2) the presence of a social partner previously paired with response-contingent cocaine failed to reinstate responding after a period of abstinence; and (3) non-reinforced responding during the reinstatement test was greater in the presence of a cocaine partner than in the presence of an abstinent partner. The observation that responding was significantly greater during the reinstatement test in the presence of a cocaine partner suggests that test rats were able to discriminate between the two partners and responding was under stimulus control; however, the excitatory effects contributed by the cocaine partner (i.e., the S^+^ discriminative stimulus) were not sufficient to reinstate responding to levels above those observed at the end of extinction. Consequently, the hypothesis that a social partner predictive of response-contingent cocaine would reinstate responding was not supported.

A growing body of evidence reveals that social variables can influence the reinstatement of drug-seeking behavior after a period of abstinence. For instance, exposure to a social threat (Lo Iacono et al., 2016) or to social defeat (Bruchas et al., 2011; Titomanlio et al., 2013) reinstates a cocaine-induced conditioned place preference in mice and rats. Similarly, exposure to an olfactory cue that predicts social defeat reinstates cocaine-seeking responses in an operant task (Manvich et al., 2016). Social defeat also increases the vulnerability to cocaine-primed reinstatement in operant procedures; however, a brief, positive social interaction decreases vulnerability to cocaine-primed reinstatement under identical conditions (Ribeiro Do Couto et al., 2009). Consistent with the latter finding, the opportunity to play with a social partner prevents the reacquisition of a cocaine-induced conditioned place preference (Fritz et al., 2011).

In the present study, two social partners served as discriminative stimuli. One partner was explicitly paired with response-contingent cocaine (i.e., the cocaine partner; S^+^) and one partner was explicitly paired with extinction (i.e., the abstinent partner; S^−^). Previous studies using both discrete and contextual stimuli as excitatory/inhibitory discriminative stimuli have reported that excitatory discriminative stimuli reliably and robustly reinstate drug seeking but inhibitory stimuli do not. For instance, passive contextual cues (e.g., distinct odor, continuous white noise) that predict cocaine availability reinstate cocaine seeking, whereas passive contextual cues that predict extinction do not (Weiss et al., 2000; Alleweireldt et al., 2001). These effects are observed whether the passive contextual cues are presented alone or with additional response-contingent cues (e.g., response-contingent houselight illumination) as part of a compound stimulus signaling cocaine availability (Ciccocioppo et al., 2002). Moreover, a passive contextual cue paired with extinction can later inhibit the reinstatement of responding produced by a cocaine prime (Mihindou et al., 2013). Collectively, these data indicate that environmental cues can produce both excitatory and inhibitory effects to control responding in reinstatement procedures.

It is important to note that the two social partners were engaging in very different behaviors, and we do not know whether it was the partner *per se* or the behavior of the partner that served as the discriminative stimulus to influence responding. We choose this particular experimental design for two reasons. First, our prior work indicates that the behavior of a social partner (i.e., whether or not that partner is self-administering cocaine) is critically important in determining the effects of social contact on measures of drug-seeking behavior. Specifically, cocaine intake is increased in the presence of a partner self-administering cocaine and decreased in the presence of a partner without access to cocaine (Smith, 2012; Smith et al., 2014). Second, we attempted to create an ecologically valid model of the social environment in which a partner that consistently uses drugs likely predicts that drug-seeking responses will be reinforced, whereas a partner that consistently practices abstinence likely predicts that drug-seeking responses will not be reinforced. Future studies will have to determine whether it was the partner or the behavior of the partner that ultimately controlled drug-seeking responses.

As noted above, we failed to find evidence that a social partner self-administering cocaine and predictive of cocaine availability significantly reinstated responding. Although few parametric manipulations were performed, we selectively choose experimental parameters that were most likely to be effective in our assay. The dose of cocaine selected (0.5 mg/kg/infusion) is a moderate dose of cocaine that engenders high rates of responding in rats (Baird et al., 2000). We previously obtained robust degrees of cocaine-primed and cue-induced reinstatement after training with this dose (Smith et al., 2012), although other investigators have reported equally robust effects with lower doses (Bespalov et al., 2000; Goeders et al., 2009). The 20-day period of training is similar to those used in previous studies reporting robust reinstatement of responding following extinction (Anderson et al., 2006; Anker et al., 2009). Throughout training, test rats responded with each partner over five consecutive days because that time frame is sufficient: (1) for rats to develop a preference for another rat with a similar history of cocaine self-administration (Smith and Pitts, 2014); and (2) for rats within social dyads to develop similar patterns of cocaine-maintained responding on a fixed-interval schedule of reinforcement (Lacy et al., 2014). The alternating and counter-balanced schedule used during training was selected to control for the potential influence of primacy or recency effects that might confer more associative strength to one partner over the other. This design also has translational appeal because social partners that signal the availability (or absence) of drugs are typically encountered repeatedly (but not continuously) during periods of occasional drug use. The 7-day extinction period is similar to that used in previous studies (Rogers and See, 2007; Feltenstein et al., 2009; Vranjkovic et al., 2014) and was sufficient for responding to plateau in the present study (Figure 1). Finally, the parameters used in the reinstatement test were similar to those we have used previously to successfully reinstate cocaine-seeking behavior with an audiovisual cue and a cocaine prime (Smith et al., 2012). Given these considerations, we therefore conclude that a social partner (and/or the behavior of a social partner) is less effective than other types of stimuli at reinstating cocaine-seeking behavior following extinction.

It is notable that the responding of cocaine partners, which had access to cocaine throughout the duration of the study, was not influenced by the presence or absence of the test rat. In a series of studies, we reported that cocaine self-administration is influenced by the behavior of a social partner, and in all those studies, responding was greater in the presence of a self-administering partner than in the presence of an abstinent partner (Smith, 2012; Smith et al., 2014; Robinson et al., 2016). An isolated control group was included in all our previous studies, and the cocaine intake of the control group was typically intermediate to that of the other social groups. Importantly, those previous studies employed between-subjects designs, and social conditions never varied within a study. It is possible that the responding of the cocaine partners in the present study would have been influenced by the partner manipulations if longer periods of isolation/social housing had been employed.

In conclusion, we were not successful at demonstrating that a social partner self-administering cocaine and predictive of response-contingent cocaine could reinstate cocaine-seeking behavior after a period of abstinence. On the other hand, we were able to demonstrate that a social partner self-administering cocaine could serve as a discriminative stimulus. This latter observation was evidenced by the differential responding observed during the reinstatement tests when the response-reinforcer contingencies were identical in the presence of the two partners. During this test, responding was significantly greater in the presence of the cocaine partner (S^+^) than the abstinent partner (S^−^), indicating that social partners can serve as discriminative stimuli to produce either excitatory or inhibitory effects on drug-seeking behavior.

## Author Contributions

MAS conceived of the study and was responsible for the experimental design. HZ and AMR conducted the study and collected the data. All authors read and approved the final manuscript.

## Conflict of Interest Statement

The authors declare that the research was conducted in the absence of any commercial or financial relationships that could be construed as a potential conflict of interest.
